# Serum Levels of MicroRNA-206 and Novel Mini-STR Assays for Carrier Detection in Duchenne Muscular Dystrophy

**DOI:** 10.3390/ijms17081334

**Published:** 2016-08-13

**Authors:** Mónica Alejandra Anaya-Segura, Héctor Rangel-Villalobos, Gabriela Martínez-Cortés, Benjamín Gómez-Díaz, Ramón Mauricio Coral-Vázquez, Edgar Oswaldo Zamora-González, Silvia García, Luz Berenice López-Hernández

**Affiliations:** 1 Center for Research and Assistance in Technology and Design of the State of Jalisco (CIATEJ, A.C.), Guadalajara 44270, Mexico; monica207383614@gmail.com; 2 Asociación de Distrofia Muscular de Occidente A.C., Guadalajara 44380, Mexico; 3 Centro Universitario de la Ciénega, Universidad de Guadalajara, Ocotlán 47820, Mexico; hrangel@cuci.udg.mx (H.R.-V.); gaby_ed15@yahoo.com.mx (G.M.-C.); 4 National Center for Research and Care in Sports Medicine, National Institute of Rehabilitation, México City 14389, Mexico; bngomez@inr.gob.mx; 5 Section of Postgraduate Studies and Research, Superior School of Medicine, National Polytechnic Institute, México City 11340, Mexico; rcoral@ipn.mx; 6 Centro Universitario del Norte, Universidad de Guadalajara, Colotlán 46200, Mexico; edgar.zamora@cunorte.udg.mx; 7 Servicio de Investigación Clínica, Centro Médico Nacional “20 de Noviembre”, Instituto de Seguridad y Servicios Sociales de los Trabajadores del Estado, México City 03100, Mexico; rolasil@yahoo.com.mx; 8 División de Investigación Biomédica, Centro Médico Nacional “20 de Noviembre”, Instituto de Seguridad y Servicios Sociales de los Trabajadores del Estado, México City 03100, Mexico

**Keywords:** microRNA-206, mini-STR, carrier detection, DMD, liquid biopsy

## Abstract

Duchenne Muscular Dystrophy (DMD) is an X-linked neuromuscular disorder in which the detection of female carriers is of the utmost importance for genetic counseling. Haplotyping with polymorphic markers and quantitation of creatine kinase levels (CK) allow tracking of the at-risk haplotype and evidence muscle damage, respectively. Such approaches are useful for carrier detection in cases of unknown mutations. The lack of informative markers and the inaccuracy of CK affect carrier detection. Therefore, herein we designed novel mini-STR (Short Tandem Repeats) assays to amplify 10 loci within the DMD gene and estimated allele frequencies and the polymorphism information content among other parameters in 337 unrelated individuals from three Mexican populations. In addition, we tested the utility of the assays for carrier detection in three families. Moreover, given that serum levels of miR-206 discern between DMD patients and controls with a high area under the curve (AUC), the potential applicability for carrier detection was assessed. The serum levels of miR-206 of non-carriers (*n* = 24) and carriers (*n* = 23) were compared by relative quantitation using real-time PCR (*p* < 0.05), which resulted in an AUC = 0.80 in the Receiver Operating Characteristic curve analysis. In conclusion, miR-206 has potential as a “liquid biopsy” for carrier detection and genetic counseling in DMD.

## 1. Introduction

Pathogenic variants within the DMD cause a spectrum of recognizable phenotypes known as dystrophinopathies. Duchenne Muscular Dystrophy (DMD) is the most severe and affects male newborns in almost all cases [1]; since it is an X-linked recessive disorder, carrier detection is of the utmost importance for genetic counseling. Female relatives require genetic testing and genetic counseling to avoid the birth of affected children without enough knowledge of the disease to make informed decisions [2]. In addition, manifestations of dystrophinopathies are present in a range of 8%–40% of heterozygous females, and this affects their life quality [3,4]. About 8% of them manifest clinically relevant features of the disease such as abnormal gait, calf hypertrophy, cardiomyopathy, cramps, exercise intolerance, and intellectual and learning disabilities, among other signs and symptoms [5].

Therefore, the Muscular Dystrophy Surveillance Tracking and Research Network (MD STARnet) has established clinical guidelines to support definite diagnosis. A definite case of dystrophinopathy should be pursued prior to carrier detection. Precise diagnosis requires a combination of clinical data, family history (FH), creatine kinase (CK) levels, genetic analysis (direct/indirect) [2], and in some cases, immunostaining of muscle biopsies [6]. Consequently, genetic counseling for at-risk females and prenatal diagnosis remain challenging for cases in which deep intronic mutations in the DMD are difficult to detect even with next-generation sequencing which often covers only exonic regions [7,8]. Indirect tracking of the haplotype that co-segregates with the disease within a family is required in those cases [2,6]. For that reason, improvements in the integration of data derived from segregation analyses with informative DNA markers such as short tandem repeats (STRs) or single nucleotide polymorphisms (SNPs), the measurement of serum biomarkers such as CK and the consideration of familiar structure allow reliable calculation of recurrence risks with specialized software such as RiscalW [8]. However, limitations of such approaches are: (1) Low heterozygosity affects segregation analysis, since the detection of different alleles between affected and unaffected males allows the indirect detection of the at-risk haplotype. Thus, the number of alleles and their frequency within a population are relevant parameters that affect carrier detection using indirect analysis and haplotyping; (2) Degraded DNA samples are challenging to amplify with conventional amplicons [9]; (3) CK levels are not optimal biomarkers for carrier detection (diagnostic efficiency = 50%) [10]. Therefore, exploration of alternative non-invasive biomarkers for carrier detection and the use of optimized multiplex amplicons represent an attractive option that may in turn outperform the above mentioned approaches. In this regard, it was recently shown that a small group of microRNAs called “dystromirs” miR-1, miR-133, and miR-206 are valuable diagnostic biomarkers for DMD because they are able to distinguish among DMD and non-affected males; in particular, the skeletal muscle-specific miR-206 is upregulated in satellite cells following muscle injury [11,12]. Interestingly, miR-206 showed higher AUCs (Area Under the Curve) to differentiate not only DMD vs. normal individuals, but also Becker Muscular Dystrophy (BMD) (a less severe type of dystrophinopathy) [11] and DMD phenotypes compared to the other microRNAs. Hence, since female DMD carriers may have subclinical muscle involvement [13], quantitation of serum levels of miR-206 may harbor potential for carrier detection. For that reason, the aims of this work were the following: (1) designing novel mini-STR assays and estimating the polymorphic information content (PIC) values in three Mexican populations; (2) performing haplotype analysis in DMD families; and (3) testing differences in serum levels of miR-206 between healthy females and DMD carriers to test its diagnostic potential for carrier detection. Cell-free nucleic acids, which include cell-free DNA, cell-free mRNA, and circulating miRNAs, are being explored for various clinical applications, such as “liquid biopsy” and “non-invasive prenatal testing” (NIPT) [14]. The approaches presented herein harbor high potential to extend the above-mentioned clinical applications for carried detection in DMD.

## 2. Results

### 2.1. Population Analysis

**Figure 1:**
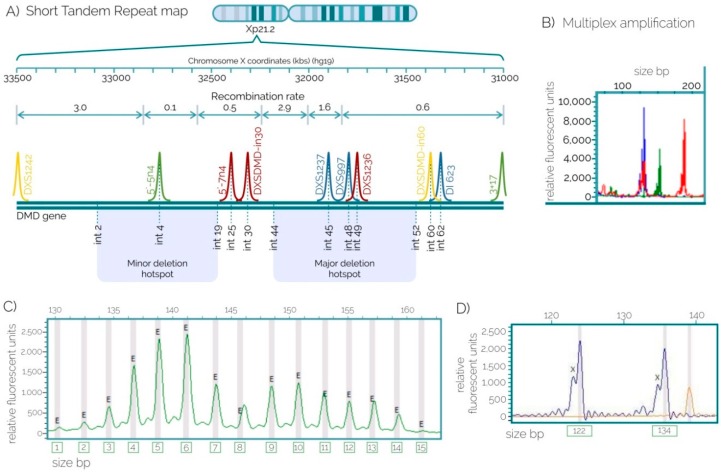
Mini-STR assays. (**A**) Simplified map of the *DMD* gene showing the location of the 10 intragenic mini-STRs, which are evenly distributed to cover the *DMD* gene from the 5′ to 3′ regions, markers are depicted with their corresponding labeled primers: NED (yellow), PET (red), VIC (green), or FAM (blue); (**B**) Amplification of one of our novel mini-STR assays. Differentially labeled primers allow the analysis of different targets in the same channel; (**C**) Genetic diversity was analyzed based on allele frequencies of 10 STRs in the three Mexican population samples (Table 1); for instance, a total of 15 different alleles were detected for the 5′-5n4 marker in these population samples; (**D**) Example of the genotype of a female for the marker DI623.

Most present-day Mexicans denominated “mestizos” as a population group, are the result of the ancestral admixture of different populations (Europeans, Amerindians and Africans). This process did not occur equally through all the geographical regions of Mexico; such heterogeneity in allele frequencies prevails nowadays [15]. This fact should be considered for genetic studies aimed at detecting candidate genes for medical traits as well as for applications of genetic variations such as the use of STRs for the detection of the at-risk haplotypes in DMD [16]. Therefore, genetic diversity was analyzed based on allele frequencies of 10 STRs within the *DMD* in the three Mexican population samples (Table 1); for instance, 15 alleles were found for the 5′-5n4 marker (Figure 1C). The markers presented a wide diversity of alleles, where at least four alleles were found for DXS1234 and up to 15 were found for DI623 (Table 1). The north region was in Hardy-Weinberg equilibrium (HWE) for all the analyzed *loci*, whereas the west and southeast regions presented HWE deviations (*p* < 0.05) for DXS1237, 5′-7n4 and DXS1234, DXS1237, and 5′-5n4 markers, respectively (Table 1). However, after applying the Bonferroni correction these deviations were not significant (*p* > 0.005). 

On the other hand, an indicator of the utility of the STRs for segregation analysis is the PIC value; which defines the expected fraction of informative offspring from one particular type of human pedigree (Table 2). Interestingly, although in most cases PIC values were similar between the studied populations, the north region showed elevated values in all STR *loci* (PIC > 0.60). PIC values observed for the 10 STRs in the three Mexican populations ranged from 0.52 to 0.88, whereas DXS1236 and DI623 were the most informative markers with more alleles (Table 1). Although in other populations similar PIC values were observed with respect to the Mexican regions studied herein, some differences were noticed as indicated in Table 2. 

### 2.2. STRs for Carrier Detection

**Figure 2:**
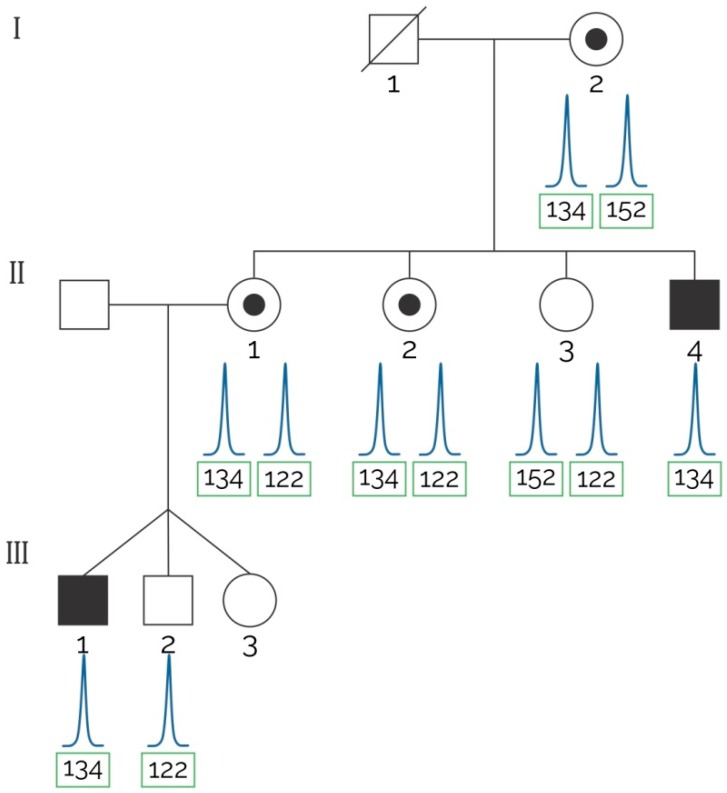
Segregation analysis in a pedigree with family history of the disease.

Seven females belonging to three unrelated families requested the result of the genetic testing derived from our research study (Table 3), and their samples were used to perform an indirect haplotype analysis. While half of the analyzed markers were conclusive for two families, seven STRs were conclusive for the remaining. The DXS1236, DI623 and DXSDMD-In30 markers were determinant in all three families, even when DXSDMD-In30 showed a PIC smaller than 0.5. The markers 5’-5n4, 5′-7n4 and DXS1237 were determinant in the first two cases, and DXSDMD-In60 was determinant exclusively in case number 3. One female carrier was detected (Figure 1D and Figure 2), while the remaining six were not carriers (Table 3).

The DI623 marker was used in a DMD case (index case II-4) with a positive family history (Figure 1D). Three sisters of the index case (II-1, II-2 and II-3) were tested and compared to the grandmother (I-2) who was an obligate carrier; she had a family history of DMD and an affected son (II-4). The older sister gave birth to non-identical triplets and the boys were tested at six years old. One of them was affected and one was healthy; the girl was considered too young to be tested for carrier detection due to ethical issues.

### 2.3. Quantitation of Serum Levels of MicroRNA-206 for Carrier Detection

**Figure 3:**
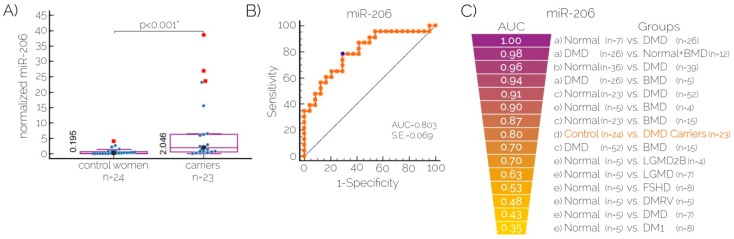
Serum levels of MicroRNA-206 for carrier detection. (**A**) Serum levels of miR-206 between DMD carriers and healthy females. The median levels are depicted with (●) and the horizontal lines bisecting the purple box plot show the interquartile range, individual values of samples are shown in (●). The most extreme values are indicated as (■); (**B**) ROC (Receiver Operating Characteristic) curve for miR-206 to classify true DMD carriers, the optimal cut-off is indicated by (●); (**C**) Heat map of AUC (Area Under the Curve) values (best values are shown on top and purple) for miR-206 to classify different phenotypes reported by others (black text and lower-case letters) and the AUC of miR-206 obtained in our study (orange text, (d)) (a) [11]; (b)[23]; (c) [12]; (d) Present study and (e) [24].

After the comparative analysis between carriers and non-carriers, miR-206 was enriched in the carriers group (Figure 3A). Serum levels of miR-206 are able to classify true DMD carriers and healthy females (Figure 3B), with an association criteria >0.45; with this number, the AUC in our study (Figure 3C) reached a value of 0.803, a standard error (SE) of 0.065, sensitivity = 78.26%, and specificity = 70.83%, with a *p*-value < 0.0001 *, whereas the positive predictive value (PPV) was 78.26 and the negative predictive value (NPV) was 99.69.

## 3. Discussion

A general definition of a biomarker is: “A characteristic that is objectively measured and evaluated as an indicator of normal, pathogenic and biological processes or pharmacological responses to a therapeutic intervention” [25]. Therefore, research to identify non-invasive biomarkers for conditions such as dystrophinopathies is a growing field nowadays. In the specific case of DMD it has been shown that levels of miR-206 positively correlate with scores of the North Star Ambulatory Assessment (NSAA), which is an endpoint to evaluate the ability of DMD patients to perform common activities [26]. Therefore, biomarkers able to detect subtle changes among related phenotypes are of particular interest, as is the case of female carriers that present almost exclusive sub-clinical muscle involvement. The field of cell-free RNAs and specifically miRNAs has generated a growing interest, because some of these miRNAs reflect the physiological status of a disease from a non-invasive method of sampling [27]. Therefore, at least for carrier detection, miR-206 and other biomarkers present in serum are of utmost importance, since dystrophin abnormalities in muscle tissue of DMD carriers are not associated with clinical variables; only 26% of non-manifesting carriers have dystrophin-negative fibers in muscle biopsies [13] which are considered invasive. Therefore, the use of the “liquid biopsy” approach may potentially outperform the use of biopsies obtained directly from muscle tissue in DMD patients, but could probably gain more relevance for carrier detection.

According to Rao et al., tests for detecting true DMD carriers must satisfy the following conditions: (a) to be positive in affected boys; (b) to not produce false negative results; (c) testing carried out using a significant number of carriers and an equal number of age- and sex-matched controls; and (d) results should be compared with an established test [28]. In this regard, a recent study of proteomics explored proteins in 16 serum samples of asymptomatic DMD carriers showing that CA3 (Carbonic anhydrase III), MDH2 (Malate Dehydrogenase 2), MYL3 (Myosin Light Chain 3), ETFA (Electron Transfer Flavoprotein Alpha Subunit) and TNNT3 (Troponin T3, Fast Skeletal Type) proteins were decreased in females compared to DMD patients treated with steroids, [29]. In addition, m-calpain from platelets was recently proven useful for identifying true DMD carriers with 90% of informative tests [28], as well as other proteins such as GDF-8 (myostatin), AUC = 0.706 (*p* = 0.028), and FSTN (follistatin), AUC = 0.877 (*p* < 0.0001) proposed by our group as biomarkers for carrier detection in DMD [30]. Nevertheless, the isolation of platelets for m-calpain detection is laborious and the ELISA technique for measuring protein concentrations may imply cross-reactivity antibody concerns for particular proteins. Therefore, herein we investigated, as an alternative, the possible use of miR-206 as a biomarker for carrier detection in DMD. Previous studies showed that miR-206 expression is muscle-specific, it is produced by myoblasts and correlates with disease progression in DMD [31], it distinguishes between DMD and BMD [12], and it increases when muscular damage occurs; therefore, miR-206 is thought to be linked to muscle repair [12,32]. Hence, we hypothesized that it may be increased in female DMD carriers. We explored the potential applicability of the isolation of miR-206 from human serum samples and its amplification by real-time PCR for carrier detection in DMD because, to our knowledge, this application was not previously explored. We did not find any correlation of miR-206 with age or body mass index (BMI) in any group. In addition, GDF-8 and FSTN did not correlate with miR-206 in this cohort. Interestingly, there were five cases with high levels of the microRNA within the carrier group, and these were all obligate carriers, but manifestations such as weakness were not reported by them, with the exception of the samples with the highest and the third-highest values of all. Those samples correspond to the mother of family 3 (Table 3) (with severe cardiac involvement) and her older daughter, the mother of the index case, a male children with a severe DMD phenotype who lost independent walking at five years old and needed assistance for walking at the age of four (the patient underwent steroid treatment for a short period of time). Cardiac involvement in the mother of the above-mentioned patient is still unknown.

**Figure 4:**
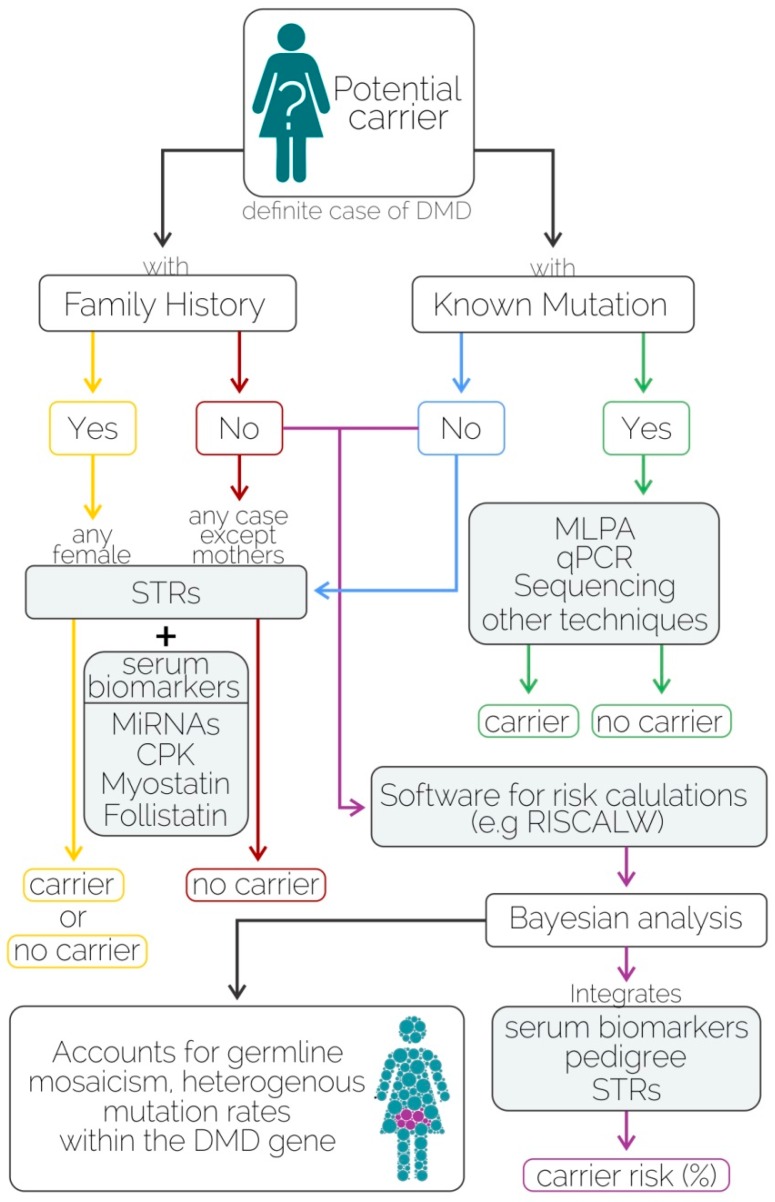
Algorithm for carrier detection using mini-STR assays and serum biomarkers.

Our data suggest that miR-206 allows the detection of DMD female carriers (who are, in most cases, asymptomatic), which demonstrates advantages over to CK as biomarker. For instance, CK decreases when muscle mass is highly diminished, even in male patients (advanced disease state) whose phenotype is more evident compared to females (usually asymptomatic), and it is altered by several conditions such as physical activity, race, age and gender [33]. In fact, CK provides a diagnostic efficiency = 50% [10], PPV = 100%, NPV = 33.3%, specificity = 100%, and sensitivity = 33.3% [10]; the latter is lower than the sensitivity displayed by miR-206 (78.2%) reported herein. Although further studies are required to confirm our findings (e.g., including larger cohorts with manifesting carriers), we demonstrated the utility of combining mini-STR assays and serum microRNAs as biomarkers to improve carrier detection and genetic counseling (Figure 4), especially in complex cases with unknown mutations and a negative family history.

In addition, our mini-STR assay allowed the amplification of multiple targets, and the study of alleles present in our population revealed the best markers for segregation analyses in Mexican families. It should be noted that best practices for genetic testing in DMD suggest that at least three DNA markers should be used to appropriately flank the gene [34], in order to avoid overlooking the recombination events that may affect genetic counseling when the location of the mutation is unknown (Figure 1). Agreeing with these recommendations, our assays included 10 loci evenly distributed along the gene, and smaller amplicons (mini-STRs) instead of the longer ones to improve the probabilities of success in analyzing highly degraded DNA samples. Most STR markers were highly polymorphic in the populations studied herein, suggesting a high level of informativeness of the markers, since the linkage between two variable loci is based on its heterozygosity. With this argument, it is widely known that there is high degree of substructure between Mexican populations that may underlie important biomedical phenotypes [16].

Although recent publications regarding the “liquid biopsy” approach are mainly focused on cancer, the use of miR-206 obtained from the serum for carrier detection in DMD as the approach reported herein could be considered a clinical application of liquid biopsies to support the diagnosis of neuromuscular disorders such as DMD. The applicability of the mini-STR assays described in this work to amplify cell-free DNA requires further exploration, although other authors have previously shown the successful amplification of 19 STR markers from maternal plasma for NIPT using mini-STR assays [35]. 

Integration of the analysis of microRNAs and DNA markers may improve carrier detection and genetic counseling in DMD. Therefore, in the present work, new tools to improve carrier screening in DMD through “liquid biopsy” approach are proposed. 

## 4. Materials and Methods

### 4.1. Sampling, DNA Isolation and Quantification

Potential carriers belonging to DMD families were referred to our laboratory between 2010 and 2015. All cases correspond to female relatives of a DMD patient diagnosed according to MD STARNET criteria [6]. Diagnosis was established considering patients clinical evaluation, which included proximal and/or distal weakness, positive Gowers maneuver, age at onset, CK levels and family history [30]. Written informed consent was obtained from all participants; this study was approved by the Institutional ethics and research committees. Control group had a mean age of 34.65 ± 12.47 (18 min–56 max) whereas the DMD carrier group showed a mean age of 40.77 ± 13.79 (17 min–86 max). There were no differences in age and BMI, between groups. Medical records of participants and clinical inspection included the following data: presence of endometriosis, cancer (any type), arthritis, active infections at the time of sample collection, osteoporosis/fractures, hepatitis and/or cirrhosis, positive cases were excluded of the analysis.

Nucleic acid isolation was performed as follows: Genomic DNA was extracted from 5 mL peripheral lymphocytes using the CTAB-DTAB method [36].

In order to gain insights regarding the allele diversity in our country, we analyzed Mexican population samples from different geographic regions, as follows: (1) North region (*n* = 105), including individuals from Chihuahua, Sinaloa, and Sonora states; (2) West region (*n* = 101), represented by the Jalisco state; and (3) Southeast region (*n* = 131), including volunteers of the Yucatán state. A Nanodrop ND-1000 spectrophotometer (Thermo Fisher Scientific, Wilmington, DE, USA) was used to measure DNA sample concentration. One hundred and five ng of DNA were used to perform MLPA and STR assays, respectively. On the other hand, human serum samples from female relatives of patients underwent purification of total RNA including miRNA with the miRNeasy Mini Kit (©QIAGEN GmbH, Hilden, Germany).

### 4.2. Mutation Detection by MLPA

Genetic screening for copy number variations within the DMD gene was done in order to confirm carrier status using MLPA technique, according to manufacturer’s instructions (P034/P035, MRC-Holland, Amsterdam, Netherlands). Data were analyzed using GeneMarker v1.91 software (© 2016 SoftGenetics, LLC., State College, PA, USA) as previously described [37].

### 4.3. Mini-STR Assay Design and Genotyping

Primers were designed according to standard conditions for multiplex mini-STR assays, which are “reduced-size STR amplicons” that outperform conventional amplicons when amplifying degraded DNA [38]. Reverse primers were labeled with different fluorescent dyes. Conditions for each assay are shown in Table 4. DNA samples were genotyped for the markers: DXS1236, DI623, 5′-5n4, 5′-7n4, DXS1237, DXS997, DXSDMD-In60, DXSDMD-In30, DXS1242 y DXS1234 at the DMD, using fluorescent polymerase chain reaction (PCR) (Figure 1A). Standard cycling was performed in a thermal cycler (Bio-Rad Laboratories, Inc, Berkeley, CA, USA). STR genotypes were obtained by fluorescent capillary electrophoresis with the ABI 310 Genetic Analyzer (Applied Biosystems, Carlsbad, CA, USA). About 1.2 μL of PCR product, 9 μL of Hi-Di formamide (Applied Biosystems), and 0.3 μL of Genescan-500 LIZ size standard (Applied Biosystems) were mixed and loaded into capillaries with POP-4 (Applied Biosystems) as separation matrix. Finally, alleles were assigned by GeneMarker HID Human STR Identity software (© 2016 SoftGenetics, LLC., State College, PA, USA) (Figure 1B).

### 4.4. Quantitation of MicroRNAs by Real Time PCR

MicroRNAs were isolated from 200 µL of human serum samples of participants using ©QIAGEN^®^ miRNeasy kit following manufacturer’s instructions. The amount of total RNA was measured with a Nanodrop ND-1000 spectrophotometer (Thermo Fisher Scientific, Wilmington, DE USA). Using microRNAs as templates, cDNA was synthesized and amplified using TaqMan MicroRNA assay for miR-206 (dystromir) and miR-223* (granulocyte-specific) (Applied Biosystems^®^); the last one was used as endogenous control, as previously reported [11]. Relative quantitation of both probes (miR-206 and miR-223*) was performed in a LightCycler^®^ 480 II (Roche, GmbH, Mannheim, Germany). Normalization was performed using Pfaffl method [39] considering true efficiency of each reaction, which is derived from serial dilutions of the target (miR-206, Efficiency, (E) = 1.845, detection limits 0.18–1.8 × 10^−3^ ng/μL) and reference gene (miR-223* E = 2.136, detection limits 0.18–1.8 × 10^−4^ ng/μL). Results were automatically calculated from the Cp values (detection threshold point) of the target and the reference amplicons.

### 4.5. Statistical Analysis

Allele frequencies and genetic diversity parameters represented by heterozygosity and PIC were estimated for each locus with the Excel spreadsheet Powerstats [40]. Exact tests to check HWE agreement for each locus were carried out using the Arlequin software [41]. This assessment was adjusted for multiple comparisons using Bonferroni correction. Values of miR-206 were tested for normality by the Shapiro-Wilk test. Statistically significant differences among experimental groups (between median values of each group) were analyzed by the Mann-Whitney U test since were non-normally distributed.

## Figures and Tables

**Table 1:** Estimation of genetic diversity and Hardy-Weinberg equilibrium in the three populations studied.

STR *loci*	North				West				Southeast			
	H_E_	H_O_	N_A_	HWE *p*-Value	H_E_	H_O_	N_A_	HWE *p*-Value	H_E_	H_O_	N_A_	HWE *p*-Value
DXS1242	0.765	0.722	8	0.5349	0.742	0.576	8	0.0581	0.713	0.848	8	0.1857
5′-5n4	0.751	0.786	12	0.9521	0.763	0.720	13	0.1182	0.578	0.442	9	0.0419
5′-7n4	0.508	0.529	5	1.0000	0.665	0.833	7	0.0127	0.566	0.769	6	0.3488
DXSDMD-In30	0.500	0.500	5	1.0000	0.503	0.423	5	0.4792	0.505	0.396	7	0.1583
DXS1237	0.815	0.615	9	0.2737	0.754	0.500	10	0.0141	0.723	0.578	11	0.0229
DXS997	0.575	0.750	5	0.2965	0.475	0.389	6	0.3778	0.373	0.333	6	0.1780
DXS1236	0.862	0.889	14	0.9764	0.800	0.969	13	0.9764	0.809	0.909	14	0.9764
DXSDMD-In60	0.652	0.667	7	0.4278	0.613	0.500	7	0.4278	0.647	0.627	8	0.4278
DI623	0.766	0.647	14	0.6746	0.776	0.613	13	0.2555	0.746	0.791	15	0.6673
DXS1234	0.675	0.389	6	0.0931	0.467	0.406	4	0.1117	0.539	0.290	5	0.0109

Expected heterozygosity (H_E_), Observed heterozygosity (H_O_), Number of alleles per *locus* (N_A_), Hardy–Weinberg equilibrium test (*p* > 0.005) (HWE).

**Table 2:** Comparison of polymorphism information content (PIC) of the 10 STRs in different populations.

STR *loci*	Mexico			Other Populations		Reference
	North	West	Southeast			
DXS1242	0.77	0.80	0.70	0.77	USA	[17]
5′-5n4	0.73	0.78	0.58	0.64	New Zealand	[18]
5′-7n4	0.62	0.69	0.62	0.52	New Zealand	[18]
DXSDMD-In30	0.61	0.56	0.63	0.65	China	[19]
DXS1237	0.82	0.78	0.73	0.89	USA	[20]
DXS997	0.67	0.58	0.53	0.70	France	[21]
DXS1236	0.88	0.87	0.88	0.93	USA	[20]
DXSDMD-In60	0.76	0.77	0.77	0.69	China	[19]
DI623	0.88	0.87	0.87	0.91	Japan	[22]
DXS1234	0.74	0.52	0.55	0.34	Japan	[22]

**Table 3:** Application of the STR assays in Mexican families. Family History (FH), Multiplex Ligation Dependent Probe Amplification (MLPA).

Family	Case Description	Applicability of STRs Indirect Haplotyping Analysis	Concluding Remarks
1	Three sisters of an index case with positive FH and two male children (triplets) were tested by MLPA and the assay proposed herein to detect the at-risk haplotype (see Figure 2).	Informative markers: 5′5n4, DXSDMD-In30, DXS1237, DXS1236, DI623.	MLPA confirmed a deletion of exons 6–7 that segregated with the at-risk haplotype traced with our assay. Two out of three sisters of the index case were carriers of the at-risk haplotype.
2	Two aunts of an index case with positive FH in which a mutation was not found after MLPA analysis solicited genetic testing.	Informative markers: 5′-5n4, 5′-7n4, DXSDMD-In30, DXS1236, DI623.	Both analyzed females were non-carriers of the at-risk haplotype; all the obligate carriers shared the at-risk haplotype found in the index case.
3	Three aunts of an index case with positive FH in which a mutation was not found after MLPA analysis solicited genetic testing.	Informative markers: DXS1242, 5′-7n4, DXS1237, DXS1236, DXSDMD-In60, DXSDMD-In30, DI623.	None of the analyzed females were carriers of the at-risk haplotype. Next-generation sequencing confirmed that a point mutation (stop codon) in exon 30 co-segregated with the at-risk haplotype.

**Table 4:** STR assay conditions for the 10 polymorphic microsatellite sites.

Marker	*Locus*	Primer Sequences (5′→3′)	Fluorophore	PCR Product (bp)	Conditions for Multiplex STRs Assays PCR		
					Denaturation	Annealing	Extension
DX997	Intron 48	AGCTGGCTTTATTTTAAGAGGACA	*FAM*	88	95 °C, 25 s	64 °C, 30 s	72 °C, 30 s
		GGGTAGCCTTCCAAGAATAGG					
DXS1237	Intron 45	GGCTATAATTCTTTAACTTTGGCAAG	*FAM*	169			
		CCACCTCTTTCCCTCTT					
DXS1236	Intron 49	CGTTTACCAGCTCAAAATCTCAAC	*PET*	111	95 °C, 18 s	62.5 °C, 20 s	72 °C, 15 s
		GGCTTTGGCCATACAGAAAA					
DI623	Intron 62	CGAGACACCCCACCTCTG	*FAM*	140			
		GCCATGGTGAATGATCAGAAA					
5′-5n4	Intron 4	GAGAGAAGGGAAAATGATGAATAAAA	*VIC*	148			
		TGTCAGAACTTTGTCACCTGTCTT					
5′-7n4	Intron 25	CTTTTAAGGCAGTTGGTGAAGC	*PET*	181			
		TCCAGGATCCAACAATATCTCA					
DXSDMD-In30	Intron 30	GTTAGTCCCTATTCTATTCCTTTC	*PET*	162	95 °C, 30 s	57 °C, 40 s	72 °C, 30 s
		AAGAATGCCACCAAAATGAC					
DXSDMD-In60	Intron 60	CGAGGGGATCAGGGTAATA	*NED*	136			
		CTGTTCTCTTCTCTGGTCATCA					
DXS1234	Region 3′	CTGTTTGCGACATTGGCTAT	*VIC*	151	95 °C, 20 s	57 °C, 40 s	72 °C, 30 s
		GCAAACATCATGGTGATAACTGA					
DXS1242	Region 5′	CAAAAATCAAATGGAAGTAGAATAGC	*NED*	112	95 °C, 30 s	59 °C, 50 s	72 °C, 40 s
		TCGCTATTCTGAAATAGTGTTTTCC					
